# Blend in or stand out: social anxiety levels shape information-sharing strategies

**DOI:** 10.1098/rspb.2022.0476

**Published:** 2022-05-25

**Authors:** Silina Zaatri, Idan M. Aderka, Uri Hertz

**Affiliations:** ^1^ Department of Cognitive Sciences, University of Haifa, Haifa, Israel; ^2^ Department of Psychology, University of Haifa, Haifa, Israel

**Keywords:** information sharing, social anxiety, social influence, social motivation, advice giving, ventral striatum

## Abstract

Although living in social groups provides many benefits for group members, such groups also serve as a setting for social competition over rank and influence. Evolutionary accounts suggest that social anxiety plays a role in regulating in-group conflict, as individuals who are concerned about social threat may choose to defer to others to maintain the hierarchical status quo. Here, we examine how social anxiety levels are related to the advice-giving style an individual adopts: a competitive influence-seeking strategy or a defensive blend-in strategy. We begin by demonstrating that similarity to others drives activity in the brain's valuation system, even during a competitive advice-taking task. Then, in three behavioural experiments, we show that social anxiety levels are related to the tendency to give advice resembling the advice given by rival advisers and to refrain from status-seeking behaviour. Social anxiety was also associated with negative social comparisons with rival advisers. Our findings highlight the role of competing social goals in shaping information sharing.

## 1. Introduction

Like other social animals, humans live in groups, upon which they are dependent for security, shelter, resources and emotional support [1,2]. Yet high-ranking members of hierarchical groups have access to more resources, making competition within the group inevitable [3,4]. Losing in such competition or being demoted or even excluded from the group can have detrimental consequences for group members. Hence, knowing when, and against whom, to compete is an important skill for group living. Recent evolutionary perspectives on competition within groups highlight the prevalent use of submissive gestures in human and non-human societies. Group members use these gestures to signal to others that they accept their lower rank and acknowledge the role of such submissive behaviours in maintaining a stable hierarchy and in minimizing conflicts [5–7]. Such accounts suggest that social anxiety may serve a regulatory role in maintaining the stability of the group hierarchy, as some group members are more focused on social threats, avoid conflicts and defer to higher ranking members [8]. Here we focus on information sharing, specifically in the form of recommendations and advice giving. This type of information sharing represents a prevalent social behaviour that has the potential to elevate the influence, status and prestige of group members over others, but also entails social risks [9–11]. We suggest that level of social anxiety affects individuals' information-sharing and advice-giving behaviour, such that those with low anxiety will use advice giving as a means of self-promotion and gaining social influence, while those with high anxiety will use advice giving defensively to minimize changes to their status, signal similarity and blend in.

The information people choose to share with others and the way in which they share it exert a major impact on group cohesion and collaboration [12,13] by enabling people to accumulate knowledge and learn from others' experience through advice and teaching [14–16]. In giving advice, advisers share their private information with others, allowing them to demonstrate their unique knowledge and influence the behaviour of others. Advice giving may therefore be a means of increasing one's influence, reflecting social status and prestige [10,11]. In previous studies, we used an advice-giving game in which participants played the role of an adviser and competed with a rival adviser for influence over an advisee's decisions. In that research, we found that advisers strategically adapt their advice giving to gain and maintain social influence [17,18]. In those studies, advisers increased their advice confidence when they were ignored by the advisee, thus making their advice stand out compared to that of rival advisers. By contrast, they gave more cautious advice when they were the preferred advisers, in line with an influence-seeking strategy from game theory [19]. Using functional magnetic resonance imaging (fMRI), we found that activity in participants' ventral striatum, an area which is part of the brain's reward and valuation system, increased when their advice was more accurate than that of rival advisers and when the advisee chose them as preferred advisers, i.e. when their influence increased [17]. This finding is in line with other observations of ventral striatum response to social rewards, such as changes in reputation [20], social comparisons [21] and social appraisal [22]. Taken together, these findings support the notion that seeking to gain social influence serves as a social motivation affecting advice giving.

Yet distinguishing oneself from others may carry social risks, as giving advice that turns out to be wrong can have negative effects on the adviser's reputation and status [23]. Advisers may therefore choose to conform to other people's opinions, passing over the opportunity to increase their influence. Conformity to other group members' opinions has been well documented in many behavioural settings [24,25]. Other people's opinions have also been shown to affect ventral striatum activity, for example, when one is exposed to items that others value [26] or when one's opinion is similar to the group's opinion [27]. These findings point to a link between reward signals in the brain and conformity. In our previous research using an advice-giving task, we observed that participants diverged from an optimal influence-seeking strategy [19]. Advisers were affected by the estimation of self-competence, i.e. the likeliness that their advice is accurate, such that they were less likely to try and change their influence level when their self-competence perception was low [17,18]. These findings indicate that the social risk associated with advice giving does not go unnoticed by advisers, and that advisers consider their likelihood of ‘winning’ when deciding whether to engage in competitive advice giving or whether to defer to the rival adviser.

Our previous findings also indicated that participants with high levels of social anxiety were generally less likely to use an influence-seeking strategy [17], indicating that advice-giving behaviour may vary among individuals along a social anxiety dimension. People with social anxiety disorder find it hard to form social interactions and perform in social contexts [28]. Subclinical modes of socially anxious behaviour are quite prevalent, making social anxiety useful as a dimension that governs individual differences in social behaviour [29]. As mentioned above, social anxiety may be related to the competitive nature of social relations, specifically in the context of rank and hierarchy and of an enhanced focus on social threat [5–8]. Consequentially, people with a high level of social anxiety are more sensitive to social evaluation and fear scrutiny by others, whether negative or positive [29–31]. In addition, social anxiety is associated with a double standard for evaluating the behaviour of others, as socially anxious individuals assume their own behaviour will be negatively evaluated while they believe that others can get away with similar behaviour [32–35]. Social anxiety, therefore, may lead to a lower likelihood of engaging in influence-enhancing behaviour such as competitive advice giving and may enhance the likelihood that they will attempt to blend in, exhibit similarity to others and avoid scrutiny [36].

Here we hypothesize that social anxiety levels are related to the balance between a motivation to stand out and gain influence and a motivation to blend in and be similar to rival advisers in an advice-giving context. We start our investigation by re-examining neuroimaging data from Hertz *et al*. [17], this time looking for the effect of advice similarity on ventral striatum activity. We then move on to replicate the findings of Hertz *et al*. [17] concerning reduced influence-seeking behaviour in participants with high levels of social anxiety. These initial steps form the basis for more direct investigation of the trade-off between blending in and standing out in competitive advice giving. We examine participants' tendency to match the advice confidence exhibited by the rival adviser by manipulating the order in which the advice is given (Experiment 1) and the average confidence of the rival adviser (Experiment 2). We then examine how this matching behaviour corresponds with levels of social anxiety. These experiments allow us to go beyond the previous observation of competition avoidance to demonstrate *active* attempts to blend in and become similar to others. Our final experiment (Experiment 3) examines whether social anxiety levels are related to more critical self-competence perception. Underestimating one's own likelihood of being correct may discourage socially anxious individuals from competing for influence and encourage them to focus more on the negative outcomes associated with being accountable for a negative outcome. Combined insights from brain activity and theory-driven behavioural experiments can demonstrate the way influence-seeking and blending-in strategies of advice-giving shift along the social anxiety dimension.

## 2. Methods

### (a) Participants

The Amazon M-Turk platform was used to recruit all participants for this study [37]. Participants were required to have a history of at least 100 approved tasks on the platform and at least 95% approval rates. All participants gave their informed consent and received monetary compensation at a fixed rate of 4 USD for 20 min of participation. Participants were not given any extra monetary incentives related to task performance, in line with previous implementation of this task [17,18]. The study was approved by the research ethics committee of the Faculty of Social Sciences at the University of Haifa, Israel (no. 038/20).

Sample size was set at about 70 participants per experiment (full details in the electronic supplementary materials). Exclusion criteria were giving the same ratings for all SPIN items, advice accuracy below 50% (i.e. participants who ignored the evidence) and using only one level of confidence in giving advice. We analysed data from 331 participants (40 excluded overall): Experiment 0 (replication): *N* = 65 (36 male, age (mean ± s.e.) 37.1 ± 11.6, age range: [19–71], 5 excluded); Experiment 1: *N* = 74 (45 male, age 37.7 ± 11.4, [20–69], 12 excluded); Experiment 2: Overconfidence condition *N* = 63 (39 male, age = 40.2 ± 10.4 [22–67], 6 excluded), Under-confidence condition *N* = 59 (33 male, age = 35.3 ± 9.7 [20–75], 7 excluded); Experiment 3: *N* = 70 (41 male, age = 37.4 ± 11.3 [21–72], 10 excluded).

### (b) Advice-giving task

**Figure 1.**
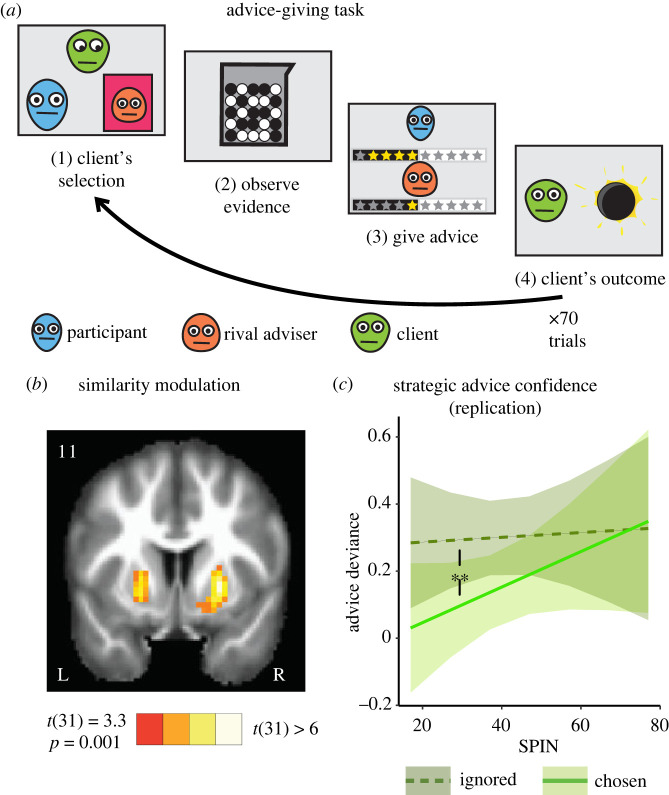
Task and replication of previous results. (*a*) Participants played the role of an adviser competing with a rival adviser for influence over a client. At the beginning of each trial, the client chose which adviser's advice he/she would follow in selecting the winning ball in the upcoming lottery (1). Both advisers then observed the rack of balls (2) and gave their advice (3). The client placed a bet according to the colour advised by the selected adviser and the outcome of the lottery was then revealed (4). A new trial then began, in which the client could switch advisers. (*b*) Ventral striatum activity increased when both advisers gave similar advice (*p* < 0.001, FWE cluster size-corrected *p* < 0.05). (*c*) Experiment 0—In a replication of previous behavioural results [17], we found that participants generally adopted an influence-seeking strategy, such that they increased their advice deviance when the client ignored them and decreased it when the client chose them. The use of this strategy exhibited a negative correlation with social anxiety levels. Lines show the estimated marginal trends from the regressions, and shaded areas represent 95% confidence of the estimation. Double asterisk (**) indicates *p* = 0.005. (Online version in colour.)

Participants played the role of advisers in an online advice-giving task (figure 1*a*) in which a client seeks advice about which coloured ball to bet on in a lottery. Two advisers compete for influence over the client's decisions. At the beginning of each trial, the client chooses the adviser whose subsequent advice will determine which coloured ball the client will bet on. From the point of view of our participants, each trial starts when the client chooses an adviser: either the participant or the rival adviser (figure 1*a*). The participant and the rival adviser are then shown the evidence in the form of a rack of black and white balls about to be entered into a raffle. The ratio between the black and white balls in the rack indicates which colour has the greatest probability of winning. The advisers then give their advice about the colour more likely to win (black/white) and estimate the likelihood this colour will win using a 5-star confidence scale. Subsequently, the advice given by the two advisers is revealed to both advisers as well as to the client. The client then bets according to the chosen adviser's advice, the winning ball is revealed to everyone, and the next trial begins, in which the client makes a new choice of adviser according the advisers' previous confidence and accuracy.

All participants played the role of adviser in the advice-giving game for 70 trials. The rival adviser's confidence was generated by algorithm, which gave calibrated confidence according to the evidence with some noise. The client's behaviour was governed by an algorithm which took the history of confidence and accuracy of both advisers into account when choosing between them (see electronic supplementary material). Both algorithms were previously used in the advice-giving task and were shown to provide results similar to those obtained with a human client and rival adviser [17]. A demo of the task is available at http://www.urihertz.net/AdviserDemo2/.

Each experiment used a different variation of the task to test a specific theoretical prediction. In Experiment 1, the order of advice was manipulated, i.e. whether the participant saw the rival adviser's advice before or after giving his own advice. In Experiment 2, the rival adviser's confidence was manipulated to be over- or under-confident by using the high (4,5 stars)/low (1,2 stars) levels of the 5-star confidence scale. In Experiment 3, the evidence in the form of the rack of black and white balls was distributed between the advisers, such that participants saw which proportion of the rack was available to the rival adviser and which was available to them.

All participants in the behavioural experiments completed the Social Phobia Inventory (SPIN) measure [38] immediately after completing the task. This questionnaire consists of 17 items related to fear (I am afraid of people in authority), avoidance (I avoid going to parties) and physical (I am bothered by blushing in front of people) aspects of social anxiety. We found high consistency for the SPIN questionnaire (Cronbach's alpha greater than 0.95 in all experiments). Participants in the fMRI experiment completed the fear of negative evaluation (FNE) questionnaire [31].

### (c) Data analysis—functional magnetic resonance imaging

All details regarding data acquisition and preprocessing can be found in Hertz *et al*. [17] (the dataset we used) as well as in the electronic supplementary material. For the analysis presented here, we used trial-by-trial variations in the absolute difference between the advice given by the participant and that given by the rival adviser as parametric modulators of activity in a general linear model (see electronic supplementary material). We examined the areas that exhibited a negative relation to the parametric modulations, i.e. areas whose activity increased when the advisers gave similar advice and decreased when they gave different advice. The complete table showing the whole-brain analysis is available in the electronic supplementary material, Table S1.

### (d) Data analysis—behaviour

In Experiments 0, 2 and 3, we used trial-by-trial advice deviance as the dependent variable in the analyses. Advice deviance was defined as the signed difference between the evidence—defined as the observed ratio of black to white balls—and the advice confidence as evaluated using the 5-star scale [17]. For example, when the observed ratio was close to 100%, an advice confidence of 5 stars was assigned an advice deviance of zero, i.e. calibrated advice. When the ratio was close to 50%, an advice confidence of 1 star was assigned zero advice deviance. Advice deviance was different from zero if the confidence rating is higher (positive deviance) or lower (negative deviance) than the probability indicated by the evidence. Average advice deviance per participants ranged between −1.5 and 1.5, with median of 0.22.

In Experiment 1, we were interested in the difference between the advice given by the participant and that given by the rival adviser. We therefore used advice difference as the dependent variable, defined as the absolute difference between the advice confidence of the player and that of the rival adviser. Average advice difference per participants ranged between 0.6 and 2.4, with median of 1.2.

We used mixed-effects linear regressions with group-level coefficients (fixed effects) to model population-level effects and individual-level coefficients (random effects) to capture average individual responses [39]. We report standardized coefficients, *t*-values and *p*-values in the main text for the effects of interest, with full details given in the electronic supplementary material. Note that standardized coefficients represent the partial correlation between the dependent and independent variables and are therefore indicators of effect size. Most of the effect sizes we report here are small (approx. 0.05), as they represent the effect within a single trial. In a within-subject design with multiple trials, such effects accumulate and lead to a significant and meaningful effect [40]. We estimated the population level marginalized means for post hoc evaluation of the effects and for post hoc comparisons [41]. All analyses were conducted using R software (R v. 4.03). Analysis packages are detailed in the electronic supplementary material.

## 3. Results

### (a) Similarity and ventral striatum activity

We sought to examine whether giving advice similar to that of the rival adviser served as a social reward in this task. We began our investigation by revisiting neuroimaging data obtained from participants performing an advice-giving task in an MRI scanner. In this previous study, we showed that two social rewards—being more accurate than the rival adviser and being chosen by the client—led to increased activity in the ventral striatum [17]. This activity was locked to the timing of social information presentation: ventral striatum activity was affected by relative accuracy immediately after the information's accuracy was revealed (the colour of the wining ball) and by client selection after the client declared the chosen adviser.

In the current study, we examined brain activity relative to advice similarity, measured as the absolute difference between the advice given by the advisers at the stage when the advice of both advisers has been revealed but the winning colour (i.e. whose advice was more accurate) has not yet been revealed (stage 3, figure 1*a*). We found that during the advice display stage, activity in the ventral striatum tracked the trial-by-trial changes in advice similarity (figure 1*b*, *p* < 0.001, FWE cluster size-corrected *p* = 0.05). Furthermore, in the previous study, we used the FNE questionnaire as a measure of social anxiety [31]. We found a correlation between the effect of similarity on activity in the left ventral striatum and participants' FNE scores (*R*^2^ = 0.2, *p* = 0.017), while activity in the right ventral striatum did not correlate with FNE scores (*R*^2^ = 0.001, *p* = 0.92). These results indicate that similarity may serve as a rewarding signal even during a competitive information-sharing task, and not just in non-competitive group conformity settings [26]. This may provide motivation for advice giving, and perhaps more so among participants with higher levels of social anxiety. Based on these neuroimaging results, we sought to take a deeper look at the effect of the motivation to provide similar advice.

### (b) Experiment 0—replication of previous behavioural results

The objective of our first experiment (*N* = 65) was to validate some adaptations we made in the experimental design and to show that the adapted design replicates the previously observed negative relation between levels of social anxiety and status-seeking strategy in advice giving [17]. In the previous study, we used the FNE questionnaire as a measure of social anxiety [31]. In the current study, we replaced this questionnaire with a broader measure, the Social Phobia Inventory (abbreviated as SPIN) [38]. We also used a shorter version of the task that included only 70 trials, compared with 130 trials in previous experiments, because it could be more easily deployed in larger cohorts of participants.

Participants completed the task online, and their advice deviance was measured (see Methods). Advice deviance was defined as the signed difference between advice confidence (number of stars) and evidence strength (percentage of same colour balls). Advice deviance was used as a dependent variable in a mixed-effects linear regression. Influence level, signified by the client's decision to ignore/choose the participant adviser at the beginning of the trial, was used as a within-subjects independent variable, SPIN score was used as a between-subjects independent variable, and the interaction between these two variables was also included in the analysis.

In line with previous studies, we found a significant influence effect (standardized coefficient = −0.08, *t*_4544_ = −4.59, *p* < 0.001), such that participants reduced their advice deviance when selected by the client. We also replicated the interaction between social anxiety levels and influence (standardized coefficient = 0.04, *t*_4544_ = 2.78, *p* = 0.005), as participants with high SPIN levels were less affected by their influence level and did not demonstrate strategic adjustment of their advice deviance (figure 1*c*). See electronic supplementary material, table S2 for full results.

### (c) Experiment 1—order of advice

**Figure 2.**
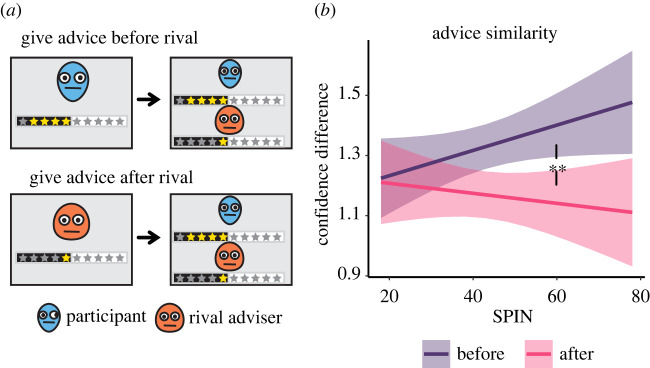
Advice similarity is related to SPIN levels (Experiment 1). (*a*) In the first variation of the advice-giving task, the order of advice giving and observation of the rival adviser's advice was alternated between trials, enabling us to examine whether participants adjusted their advice to match that of the rival adviser. (*b*) Participants with high SPIN scores made larger adjustments in their advice confidence to match the rival adviser's advice, as measured by the absolute difference between the advice confidence of the participant and that of the rival adviser. Lines show the estimated marginal trends from the regressions, and shaded areas represent 95% confidence of the estimation. Double asterisk (**) indicates *p* = 0.002. (Online version in colour.)

Our replication indicated that participants with high SPIN scores were less engaged in strategic status-seeking behaviour. This could be the result of ignoring the social aspects of the task and simply reporting the evidence they observe. Alternatively, these participants may have been attuned to the social context yet made their decision in order to match that of the other adviser rather than to gain influence. To test this hypothesis, we used a variation of the advice-giving task in which we alternated the order of advice giving and observation of the rival adviser's advice (figure 2*a*). In half of the trials, the order was the same as in the original design, such that participants first gave their advice and then observed the rival adviser's advice. In the other half, participants first observed the rival adviser's advice and then gave their advice. We predicted that participants who heeded the rival adviser's advice would adjust their own advice to resemble that of the rival adviser when this information is available, resulting in an interaction between SPIN levels and order of advice effect on advice difference.

We measured participants' (*N* = 74) advice difference, i.e. the absolute difference in advice confidence between the advice of the participant and that of the rival adviser. We then used this difference as a dependent variable in a mixed-effects linear regression. We used SPIN as a between-subjects independent variable, the order of advice and observation as a within-subjects independent variable, and the interaction between these two variables. We found a significant interaction effect (standardized coefficient = −0.04, *t*_5172_ = −3.1, *p* = 0.002), such that participants with high SPIN levels were more sensitive to order effect and gave advice that was more similar to that of the rival adviser when this advice was revealed to them prior to giving their advice (figure 2*b*; electronic supplementary material, Table S2). Note that the difference between slopes in figure 2 was significant, while each slope did not differ significantly from 0 (see electronic supplementary material). These results indicate that participants with high SPIN scores did not ignore the social context, but rather than displaying status-seeking behaviour they displayed similarity-seeking behaviour.

### (d) Experiment 2—confidence matching

The order experiment indicated that participants actively adapted their advice to match the advice of the rival adviser. To further specify this preference for similarity, we used a second variation of the advice-giving task. In this variation, participants were matched either with a consistently overconfident rival adviser who gave only highly confident advice, resulting in positive advice deviance (*N* = 63), or with an under-confident adviser who gave only advice marked by low confidence, resulting in negative advice deviance (*N* = 59) (see electronic supplementary material, figure S1). The order of advice in this task was always the same: participants were always the first to give advice, before learning the rival adviser's advice. Participants could adjust their advice confidence to match that of the rival adviser's advice by accumulating observations over time [42,43]. To test advice confidence matching behaviour across multiple conditions, we also included the data from experiment 0 (the replication experiment), in which participants faced a calibrated rival adviser whose advice confidence matched the evidence.

**Figure 3.**
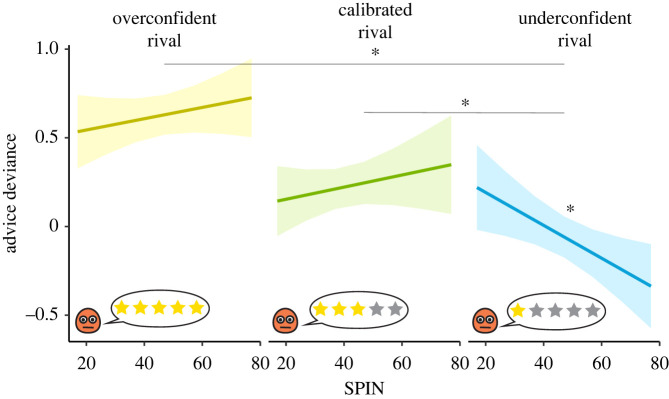
Matching rival adviser's advice confidence (Experiment 2). Three groups of participants performed the advice-giving task and were matched with an overconfident, calibrated or underconfident rival adviser. All participants matched the confidence of the overconfident and calibrated rival advisers. By contrast, matching the underconfident adviser's confidence was correlated with SPIN levels. Individuals with high SPIN levels always matched the rival adviser's confidence. Lines show the estimated marginal trends from the regressions, and shaded areas represent 95% confidence of the estimation. Asterisk (*) indicates *p* < 0.05. (Online version in colour.)

We used a mixed-effects model to investigate how advice deviance was affected by participants' SPIN levels by examining the average confidence of the rival adviser (overconfident/calibrated/underconfident) as well as the interaction between SPIN and the rival adviser's confidence. We found a significant interaction between SPIN and rival adviser's confidence in the form of a negative relation between SPIN and advice deviance when matched with the underconfident adviser (standardized coefficient = −0.21, *t*_13082_ = −2.53, *p* = 0.012). By contrast, no effect of SPIN levels was observed when participants interacted with the calibrated or overconfident rival advisers (figure 3; electronic supplementary material, table T3). In post hoc comparisons of the slopes, the SPIN effect in the underconfident rival condition differed significantly from both the calibrated rival condition (*Z* = 2.53, *p* = 0.031) and the overconfident rival condition (*Z* = 2.65, *p* = 0.022). Participants with *low* levels of SPIN matched the confidence of the overconfident and calibrated advisers, but did not decrease their confidence when matched with an underconfident rival. However, participants with *high* SPIN levels demonstrated an increased tendency to match the rival adviser's advice, matching the rival adviser's confidence in all conditions.

### (e) Experiment 3—social comparison

**Figure 4.**
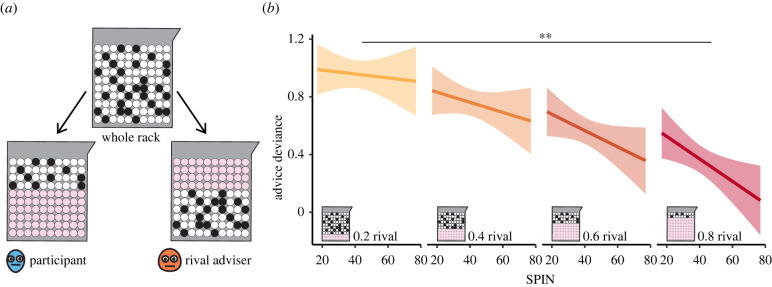
Biased social comparison effect on advice confidence (Experiment 3). (*a*) On each trial, the rack of balls was divided between the two advisers, with pink balls indicating balls available to the rival adviser. On some trials, the participant observed a larger portion of the rack of balls than the rival, and on others, the participant observed a smaller portion. (*b*) All participants exhibited a reduction in advice confidence when they observed a smaller portion of evidence than the rival. This reduction was more extreme for participants with high SPIN levels, yielding a significant interaction effect. Lines show the estimated marginal trends from the regressions, and shaded areas represent 95% confidence of the estimation. Double asterisk (**) indicates *p* < 0.005, referring to the overall interaction effect of SPIN and evidence. (Online version in colour.)

Our last experimental variation was aimed at examining a self-competence account that may be related to the over-matching behaviour of participants with high SPIN levels. We hypothesized that biased social comparison with a rival adviser, i.e. judging one's own abilities as lower than those of others, may contribute to the over-matching behaviour we observed in the previous experiments. To test this hypothesis, we used a variation of the advice-giving task in which the evidence, i.e. the rack of black and white balls indicating which colour was more likely to win, was asymmetrically divided between the advisers on each trial (figure 4*a*). We divided the evidence into four levels, ranging from 0.2 for rival and 0.8 for participant, through 0.4/0.6 and 0.6/0.4 to 0.8/0.2. Because the black and white balls were randomly distributed in the rack of balls, the *ratio* of black and white balls in each division was similar. That is, both advisers observed a similar *ratio* of black and white balls, even though they observed a different portion of the rack.

Using a mixed-effects model, we examined how advice deviance was affected by participants' SPIN levels, by the percentage of evidence available to the rival (within-subject) and by the interaction between these two factors. We found that evidence percentage exerted a significant effect on advice deviance (standardized coefficient = −0.21, *t*_7274_ = −3.91, *p* = 0.0001), as all participants tended to give less confident advice when the rival observed a larger percentage of the rack of balls than they did (figure 4*b*; electronic supplementary material, table T4). In addition, we found an interaction between SPIN and evidence percentage (standardized coefficient = −0.04, *t*_7274_ = −3.40, *p* = 0.0007). Participants with high levels of SPIN exhibited a steeper reduction in advice deviance when the rival adviser observed more evidence than they did. This result supports the notion that social anxiety level is associated with biased self-other comparison and that this aspect may further support the over-alignment effect observed in the previous experiment.

## 4. Discussion

Previous research has highlighted that advice giving is shaped by the motivation to gain influence, either in terms of behaviour or in neural activity in the ventral striatum. In the current study, we examined whether individuals with high levels of social anxiety use a defensive blending-in advice strategy as a way to avoid changes in social influence levels. First, we revisited neuroimaging data from previous work and observed that similarity to rivals elicits a response in the ventral striatum, indicating that similarity can serve as a social reward even in competitive settings, in addition to social rewards associated with being more accurate than others and increased influence [17]. We also replicated the behavioural results from our previous research, showing that social anxiety levels negatively correlated with the degree to which participants demonstrated influence-seeking behaviour (Experiment 0). We then conducted three experiments to examine how levels of social anxiety are related to advice similarity and to biased self-competence estimation. We found that the degree to which participants adjusted their advice to be similar to that of rival advisers (Experiment 1) and matched their overall advice confidence to that of a rival adviser (Experiment 2) correlated with social anxiety levels. Finally, we found that levels of social anxiety correlated with biased self-competence perception (Experiment 3). Our results show that advice giving was shaped both by the motivation to gain influence by following a strategy of standing out and promoting one's uniqueness vis-à-vis rival advisers, and by the motivation to avoid changes in social influence by following a strategy of blending in. The balance between these motivations correlated with levels of social anxiety in the general population, such that among those with high levels of social anxiety, this balance leans away from influence seeking and toward blending in.

Our main findings are that participants with high levels of social anxiety adopted fitting-in strategies more than influence-seeking strategies. This finding is in line with the use of submissive strategies to avoid social conflicts, even at the price of losing the opportunity to increase one's rank within a group [5–8]. Our study directly assesses a form of social behaviour that can promote or demote one's social influence, which is related to social rank. Our findings regarding the avoidance of behaviour that may challenge the current hierarchical structure are in line with the notion of social anxiety as a regulator of rank-related conflicts within a group [5]. In our task, a positive change in one's influence, in the form of being selected in the following trial, makes one vulnerable both to scrutiny by the client and to competition from the rival adviser. Therefore, social anxiety may lead to employing defensive mechanisms, both by avoiding negative appraisal when one makes a mistake and by reneging on positive appraisal and increased influence [44]. The observed shift towards blending in and preferring to be similar to rival advisers may be related to the shift in self-competence associated with social anxiety. Previous studies indicated that high levels of social anxiety were associated with over-reliance on negative information about one's own level of competence [32,35] and increased considerations of other people's behaviour [34]. Our findings in Experiment 3 indicate a similar bias in the context of information sharing, as participants with high levels of social anxiety were more cautious in their advice when they had less evidence than a rival adviser. In previous research, we found that social influence-seeking behaviour was modulated by self-competence perception [17,18]. Hence, biased self-competence perception may discourage socially anxious participants from engaging in competitive influence-seeking behaviour.

Our results revealed asymmetry in the way the social anxiety dimension affects advice confidence. Participants with high levels of social anxiety adapted their advice confidence to resemble that of rival advisers in the case of both overconfident and underconfident rival advisers. By contrast, participants with low levels of social anxiety adapted their advice confidence only when faced with highly confident or calibrated rivals but not with underconfident advisers. This effect is in line with accounts of self-presentation in social anxiety, which highlight a strong need to impress others and to exhibit agreement and conformity with others [36,45]. Our finding supports this perspective, showing that the motivation to blend in is not restricted to displaying timid behaviour but also applies to agreeing strongly with others, depending on the context [36].

Whereas our current study examined a very specific type of information-sharing—advice giving—our findings may be extended to other types of information sharing that share some characteristics with advice. One such characteristic is the possibility of linking the shared information (and its consequences) to the person sharing the information, which may encourage the person to be mindful about the manner and content of the shared information [45,46]. Social anxiety has been suggested to be related to self-presentation considerations and therefore may play a role in many types of information-sharing scenarios [7]. Nevertheless, it may play a smaller role in cases where the identity of the sharer is anonymous. Another factor is that the shared information is related to first-hand experience of the person sharing the information. In our case, advisers reported on evidence they themselves observed. In such cases, the outcome of the advice may be indicative of the adviser's capabilities. In many information-sharing scenarios, however, the adviser is relaying hearsay information, second-hand advice, rumours and so on, especially in the case of gossip [47,48]. In such cases, advisers may be expected to maintain different epistemic norms. That is, saying ‘I heard that…’ may make the adviser less accountable for the accuracy of the information that follows [49,50]. This reduced accountability may influence the effect of social anxiety on information sharing found in the present study.

To conclude, we showed that different motivations, whether to gain social influence or to blend in, shape advice giving and that the social anxiety dimension is related to the balance between these motivations. Our results provide evidence for the roles of motivational and information-processing factors in social anxiety, suggesting that biased self-competence perception may contribute to a shift in social goals, leaving open the role played by biased social risks and reward perception in information sharing. The approach we adopted first sought to identify different potential goals based on the literature and neuroimaging findings, and then examined these goals in specifically tailored experiments. This allowed us to better predict the conditions under which social anxiety may lead to a distinguished behavioural pattern and those in which social anxiety should not have any observable effects. Treating social anxiety as a dimension and not only as a binary factor with specific clinical implications may be useful in other studies of information-sharing style and motivations and can inform our understanding of the way information is spread in the population.

## Data Availability

All behavioural data and analysis scripts are available at: https://osf.io/bh2p6. The data are provided in the electronic supplementary material [51].
